# Study on the Physical Properties and Application of a Novel Pharmaceutical Excipient Made from Starch and Cellulose Co-Processing

**DOI:** 10.3390/ph18091389

**Published:** 2025-09-17

**Authors:** Yong Bi, Hanfang Lei, Ying Fang, Simeng Wang, Jihui Tang

**Affiliations:** Research and Industrialization of New Drug Release Technology Joint Laboratory of Anhui Province, School of Pharmacy, Anhui Medical University, Hefei 230032, China; by@shanhe01.com (Y.B.); leihf@shanhe01.com (H.L.);

**Keywords:** pregelatinized starch, microcrystalline cellulose, co-processing, pharmaceutical excipient, direct compression, dilution capacity

## Abstract

**Objective:** This article investigated the structural characteristics, powder properties, and performance variations of co-processed pregelatinized starch (PS) and microcrystalline cellulose (MCC) at varying ratios. **Methods:** Scanning Electron Microscopy (SEM) revealed the embedding of MCC within the PS matrix. Fourier-transform infrared spectroscopy (FTIR) and X-ray diffraction (XRD) analysis indicated no chemical interaction between the starch and MCC during processing. The physical properties of the co-processed materials were evaluated using multiple indicators, such as the Carr index, and their properties in pharmaceutical applications were evaluated using multiple indicators, such as tensile strength and dilution capacity. **Results:** The absence of new chemical substances during co-processing, as confirmed by FTIR/XRD analyses, coupled with SEM evidence of a physically interlocked MCC-PS architecture, conclusively demonstrates that structural reorganization occurred via physical mechanisms. An increase in the MCC proportion enhanced the tensile strength of the co-processed material while decreasing the Carr’s index, particle size, tapped density, bulk density, swelling, and water-soluble content. A co-processed sample (PS:MCC = 7:3) was selected for application in formulations. The co-processed material exhibited superior compactibility compared to a physical mixture and demonstrated favorable dilution capacity in poorly compactible model drugs, including Linaoxin and Lingzhi spore powder, as well as higher biological inertness. **Conclusions:** These findings suggest that the co-processed PS and MCC possess excellent compactibility and dilution capacity. The co-processed excipient demonstrates applicability in direct compression manufacturing of oral solid dosage forms (e.g., tablets), offering distinct advantages for high drug-loading formulations.

## 1. Introduction

Tablets are currently the most widely used dosage form in pharmaceutical manufacturing, typically produced via wet granulation or direct compression methods [1]. Compared to wet granulation, direct compression of powders eliminates the granulation process, resulting in higher efficiency, lower costs, and facilitating industrialization [2]. Furthermore, direct compression plays a crucial role in the development of newly developed formulations such as orally disintegrating tablets, fast-release tablets, and sustained-release tablets. Developed countries have already adopted direct compression for over 60% of their drug products, whereas in China, this figure is less than 20%, indicating significant development potential and prospects for direct compression in China [3]. The development of excipients plays a vital technical support role in direct compression, as the quality of excipients directly impacts the tablet’s strength, uniformity, and disintegration properties. Currently, various materials from different sources have been developed as direct compression excipients, including lactose, starch derivatives, cellulose derivatives, inorganic salts, polyols, and sugar-based materials [4]. Moreover, to overcome the drawbacks of single excipients, such as poor flowability of microcrystalline cellulose, poor compressibility of pregelatinized starch, and poor binding ability of lactose, co-processed excipients are produced using two or more components through specific processes (spray drying, co-crystallization, and fluid bed granulation) [5]. Examples include silicified microcrystalline cellulose [6] and powdered cellulose lactose [7], which exhibit good flowability and compressibility.

Pregelatinized starch (PS) is a modified starch. At the gelatinization temperature, starch granules swell, amylose leaches out, amylopectin double helix unwinds, and the crystalline structure disappears [8,9]. Currently, PS is mainly produced by extrusion puffing, spray drying, or drum drying of starch emulsion solutions. It has been reported that high-temperature and high-pressure alcohol method, alcohol-alkali method, and water-ethanol method have been developed to prepare PS [10,11]. Compared with starch, PS has significantly improved compressibility, has a certain viscosity, and is low in cost. It is often used as a diluent, binder, and disintegrant in pharmaceutical excipients [12,13]. However, PS is an elastic excipient, which easily returns to its original shape after the pressure disappears, has weak binding properties, and the tablet hardness is low [14]. Microcrystalline cellulose (MCC) is a hydrolysate of natural cellulose, and cellulose is hydrolyzed into short rod-shaped particles by dilute acid at high temperature. In direct compression materials, MCC has high compressibility [15]. However, due to its poor fluidity, it is usually used in combination with other excipients to improve fluidity [16]. The compressibility enhancement observed in co-processed systems cannot be replicated through mere physical blending of commercial pregelatinized starch and microcrystalline cellulose.

The development of co-processed materials represents a viable strategy to address the compressibility limitations inherent in pregelatinized starch systems. Relevant studies have reported that the co-processed product of hydroxypropyl starch and MCC (84:16) can prepare tablets with higher hardness [17], but starch needs to undergo hydroxypropyl modification, which is costly and not conducive to industrialization. It is noteworthy that no research has yet reported on a method for synergistically improving PS/MCC defects using unmodified PS as the primary agent through physical co-processing. Therefore, this study used PS as the primary agent and treated it with MCC through a special method to prepare a co-processed PS and MCC. Characterization of multiple physical properties and applications in three drugs (Linaoxin, Levofloxacin, and Lingzhi spore powder) confirmed their promising application prospects. This study, for the first time, attempted to utilize an “ethanol-water solvent co-processing” technique to achieve a physical intercalation (non-chemical bonding) of pregelatinized starch and microcrystalline cellulose, overcoming the high tensile strength limitations of traditional physical mixtures. This excipient provided a cost-effective solution for direct compression of oral solid dosage forms while avoiding the biocompatibility risks associated with chemically modified excipients (such as residual crosslinkers).

## 2. Results and Discussion

### 2.1. Microscopic Morphology

**Figure 1:**
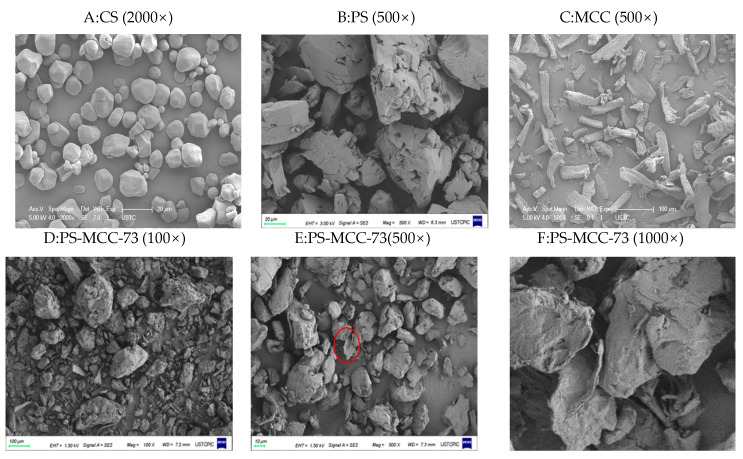
Scanning electron microscopy (SEM) (**A**) corn Starch (CS); (**B**) pregelatinized starch (PS); (**C**) microcrystalline cellulose (MCC); (**D**–**F**) different magnifications of PS-MCC-73; (**F**) shows an enlarged view of the region indicated by the red circle in (**E**).

Native corn starch granules exhibited an intact and smooth surface (Figure 1A) [18]. In contrast, pregelatinized starch (PS) prepared via heat treatment in the ethanol-water system displayed significantly roughened particle surfaces accompanied by fissure formation (Figure 1B) [19]. This phenomenon originated from the crystalline structure disruption and localized gelatinization induced by water absorption and swelling of starch within the system. Polarized light micrographs (Figure S1) also showed the pregelatinized state of starch. Within the PS-MCC co-processed material, the characteristic short rod-like morphology of microcrystalline cellulose (MCC) (Figure 1C) was notably absent (Figure 1D). This observation indicated that the preparation process achieved physical embedding of MCC within the pregelatinized starch matrix [17,19]. This embedding mechanism was consistent with the theory proposed by Parmar et al. [20] that MCC interacts with starch molecules through hydrogen bonds, forming a three-dimensional network structure. This network structure helped to restrict the mobility of starch chains and enhance the stability of the system. This structure not only inhibited excessive swelling of starch granules and leaching of amylose and amylopectin molecules, but also reduced the number of cracks and surface roughness of the PS-MCC co-processed materials (Figure 1E,F) compared to pure PS.

### 2.2. FTIR and XRD Analysis

**Figure 2:**
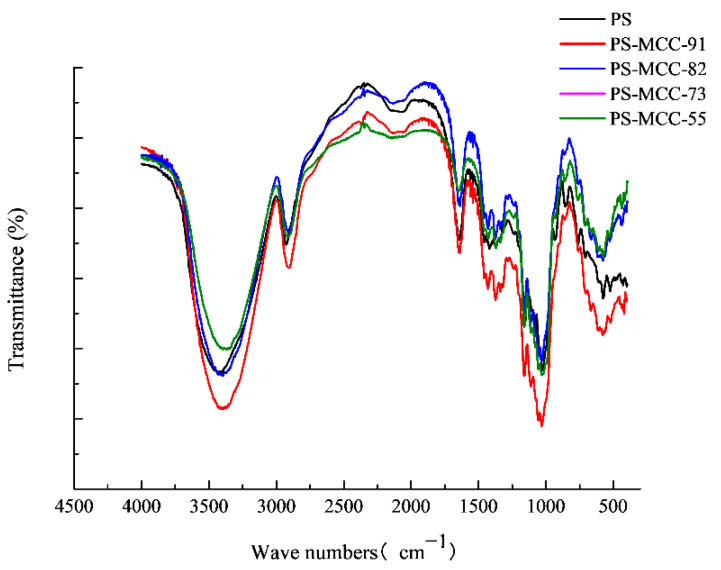
FTIR spectra of PS-MCC with different MCC contents, 91, 82, 73, and 55, represent their relative proportions.

**Figure 3:**
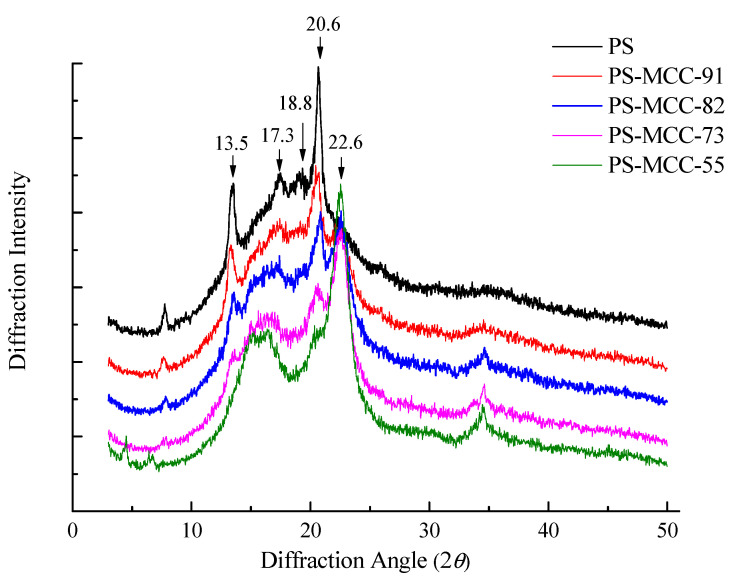
XRD of PS-MCC with different MCC contents, 91, 82, 73, and 55, represent their relative proportions.

As shown in Figure 2, both pregelatinized starch (PS) and its co-processed materials with microcrystalline cellulose (MCC) exhibit strong absorption peaks near 3400 cm^−1^, 2930 cm^−1^, and 1644 cm^−1^. These peaks are attributed to the O-H stretching vibration of hydroxyl groups, the C-H stretching vibration, and the O-H bending vibration, respectively [21,22]. The absorption peaks observed at 1162 cm^−1^, 1085 cm^−1^, and 1015 cm^−1^ are associated with vibrations of the glucopyranose ring. No new absorption peaks were observed in the co-processed pregelatinized starch-microcrystalline cellulose product. As illustrated in Figure 3, pregelatinized corn starch displays the characteristic diffraction pattern of a Type A crystalline structure, with distinct peaks at 13.5°, 17.3°, 18.8° (appearing as a doublet), and 20.6°. Microcrystalline cellulose (MCC) shows its characteristic peak for Cellulose I crystallinity at 22.6° [23]. In the co-processed PS-MCC samples, the intensity of the characteristic starch diffraction peaks diminished with decreasing starch content, while the intensity of the characteristic MCC peak increased with higher MCC content. No new diffraction peaks were detected in any of the co-processed pregelatinized starch-microcrystalline cellulose samples. These findings suggest that during the co-processing of PS and MCC, no new covalent bonds were formed between the starch and MCC, indicating that only physical changes occurred, and no chemical reactions took place [24].

### 2.3. Particle Size

Particle size and its distribution were key factors influencing powder flowability, compressibility, and tablet weight variation [25]. As shown in Table 1, the proportion of microcrystalline cellulose (MCC) exerted a significant regulatory effect on the particle size distribution of the pregelatinized starch-microcrystalline cellulose (PS-MCC) co-processed material. When the MCC proportion increased from 0% to 30%, the particle size exhibited a gradual decreasing trend. However, upon further increasing the proportion to 50%, the particle size decreased sharply, accompanied by a significant increase in the span, resulting in a broader distribution.

This phenomenon could originate from three synergistic mechanisms: Firstly, the highly crystalline MCC underwent brittle fracture under mechanical force, directly generating fine particles. Secondly, MCC embedded within the starch matrix created interfacial defects, reducing the fracture energy and promoting starch fragmentation. Finally, a 30% MCC proportion represented a critical point, where the system transitioned from “starch plasticity-dominated behavior” to an alternative regime.

This mechanism is strongly aligned with the theory proposed by Parmar et al. [20]—that brittle excipients optimize particle size distribution through fracture. It elucidated the structure-property relationship between material characteristics and processing behavior within the co-processed material.

### 2.4. Powder Flow Properties

Powder flowability is a critical factor influencing the feasibility of direct compression. The tapped density, bulk density, and Carr’s index are indicative of a powder’s compressibility and flow characteristics; a lower Carr’s index signifies superior powder flowability [22]. The Carr’s index of PS-MCC increases with the proportion of microcrystalline cellulose, indicating that microcrystalline cellulose diminishes the flowability of the co-processed material (Table 2). This finding aligns with the experimental results reported by Limwong et al. [17]. Powder flowability suitable for production typically requires an angle of repose ≤ 40°, a bulk density > 0.4 g/cm^3^, and a Carr’s index < 30% [26]. Among these, only PS-MCC-55 fails to meet these criteria, rendering it unsuitable for industrial-scale manufacturing.

### 2.5. Swelling and Water-Soluble Substances

The water-soluble content primarily comprises low molecular weight substances resulting from the starch hydrolysis during the preparation process. Under identical preparation conditions, a higher water-soluble content indicates a more pronounced starch hydrolysis reaction [27]. As demonstrated in Table 3, the water-soluble content of PS-MCC decreases with increasing proportions of microcrystalline cellulose, indicating that MCC significantly inhibits the starch hydrolysis reaction. Swelling is the primary disintegration mechanism for starch-based excipients [28]. Given that MCC lacks water absorption and swelling capacity, the swelling capacity of the co-processed material begins to decline when the proportion of MCC exceeds 20%. An excessively high proportion of MCC may potentially compromise the disintegration performance of PS-MCC.

### 2.6. Tensile Strength

**Figure 4:**
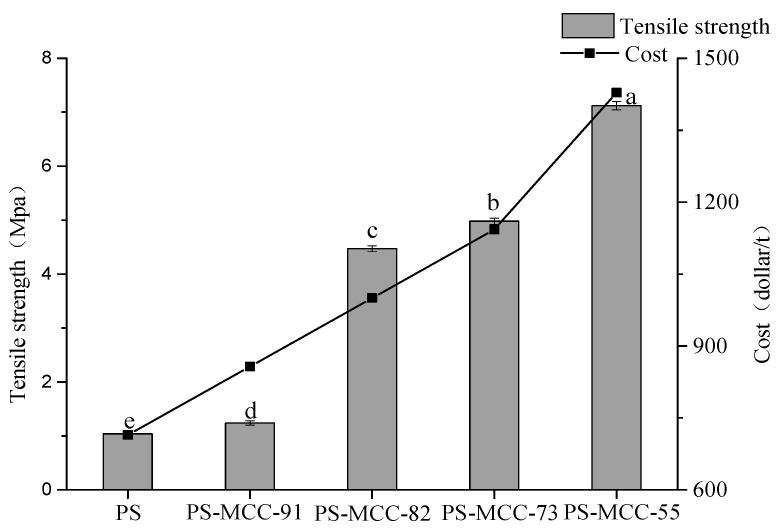
Tensile strength and cost of PS-MCC with different MCC contents, 91, 82, 73, and 55, represent their relative proportions. Different lowercase letters (a–e) indicate significant differences between different samples, *p* < 0.05.

**Figure 5:**
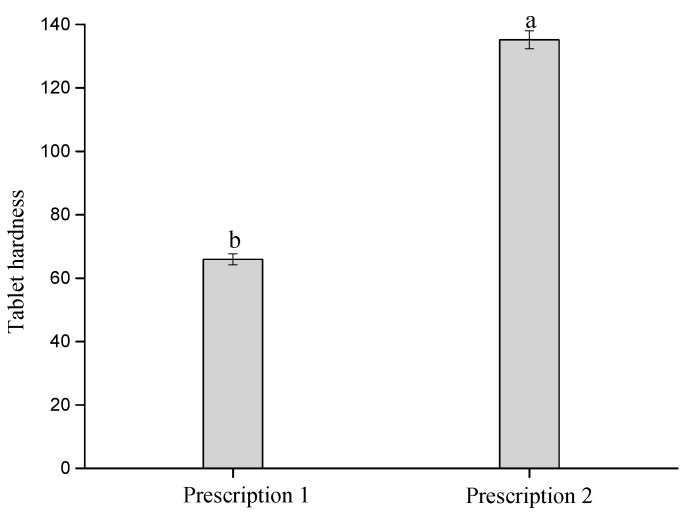
Bar graph of tablet hardness test according to the prescription in Table 5 of this study. Different lowercase letters (a,b) indicate significant differences between different samples, *p* < 0.05.

Tensile strength, which reflects the compressibility and formability of materials, is widely used in the quality evaluation and formulation screening of tablets [29]. As shown in Figure 4, PS exhibited the lowest tensile strength (1.07 MPa). The tensile strength increased significantly with the increase of the MCC ratio in the co-processed excipient of pregelatinized starch and microcrystalline cellulose. The tensile strength of PS-MCC-91 was only 1.17 MPa, but the tensile strength of PS-MCC-82 increased by 3.82 times compared with PS-MCC-91, with the largest increase. This indicates that the compressibility of the co-processed material with MCC content ≥ 20% was significantly improved. Although the higher the proportion of MCC, the higher the compressibility of the co-processed materials, PS is less expensive than MCC. Considering economic factors and good tableting performance, a ratio of starch to MCC of 7:3 is considered a suitable formulation for preparing the co-processed excipient of PS and MCC. In the Levofloxacin formulation, PS-MCC-73 and a physical mixture of commercially available pregelatinized starch SH-102 and microcrystalline cellulose SH-YJ-H were used as direct compression fillers, respectively. The Formula (6) revealed that tensile strength was directly proportional to hardness (TS ∝ F) but requires normalization by the geometric dimensions of the tablet. As shown in Figure 5, the Levofloxacin tablets compressed by PS-MCC-73 had higher hardness, indicating that PS-MCC-73 had better direct compression performance.

### 2.7. Dilution Capacity

Drug loading is a critical parameter for evaluating the quality of solid dosage forms, with dilution capacity representing the maximum drug loading capacity of an excipient for a model drug [27]. Linaoxin is an extract of traditional Chinese medicines, including Danshen, Chuanxiong, Gegen, Dilong, Chishao, Honghua, Yujin, and Zhiheshouwu, while Lingzhi spore powder is derived from the crushing of *Ganoderma lucidum* spores. Both Linaoxin and Lingzhi spore powder are categorized as poorly compressible traditional Chinese medicines. As indicated in Table 4, the co-processed excipient of PS and MCC (PS-MCC-73) exhibited favorable drug loading for both model drugs. The dilution capacity of PS-MCC-73 reached 50% and 40% for Linaoxin and Lingzhi spore powder, respectively. PS-MCC-73 can significantly enhance drug loading.

### 2.8. Biological Properties

**Figure 6:**
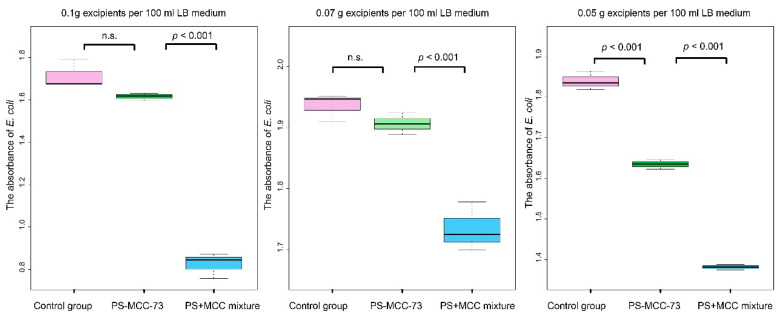
Comparison of the growth of *E. coli* in LB liquid medium under different concentrations of PS-MCC-73 and PS + MCC mixture compared with the control group. n.s. stands for statistically not significant.

In addition, to verify whether there are differences in the biological properties of PS-MCC-73 and the corresponding mixture, we placed them in LB liquid culture medium containing *E. coli*, respectively. The results showed that there were significant differences between the two in the three concentration experiments (*p* value < 0.001), and at the concentrations of 0.1 g/100 mL and 0.07 g/100 mL, the number of *E. coli* they cultured was less than that of the blank control group (without excipients), but there was no statistical significance (Figure 6). This result suggests that compared with the corresponding mixture, PS-MCC-73 will have less impact on the contacted organisms. Studies have reported that pregelatinized starch and microcrystalline cellulose have a certain inhibitory effect on *E. coli* [30,31], and their inhibitory effect on *E. coli* may be weakened after co-processing.

Pregelatinized starch and microcrystalline cellulose co-processed materials can be used as an improved direct compression excipient for pregelatinized starch. No new chemical substances were produced during the preparation of the co-processed materials of starch and microcrystalline cellulose, and MCC and PC have a physical chimeric structure. Through a comprehensive evaluation, it was found that compared with single-component pregelatinized starch, its compressibility was significantly improved, and its preparation ratio had a significant impact on its performance. In formulation applications, PS-MCC-73 (PS:MCC= 7:3) showed good compressibility and dilution potential, which can meet the needs of high-drug-loaded formulations, suggesting that it may become a promising new excipient for direct compression tableting of drugs. In addition, the intrinsic mechanism and quality standards of the pregelatinized starch and microcrystalline cellulose co-processed excipients obtained in this study need further study.

## 3. Materials and Methods

### 3.1. Instruments and Materials

The main instruments and equipment involved in this study include Antaris II Fourier transform infrared spectrometer (Thermo Fisher Scientific, Waltham, MA, USA), D2 PHASER X-ray diffractometer (Bruker AXS, Karlsruhe, Germany), and SU8100 scanning electron microscope (Hitachi High-Tech, Tokyo, Japan), etc.

Corn starch, microcrystalline cellulose (MCC), and magnesium stearate (Anhui Shanhe Pharmaceutical Excipients Co., Ltd., Huainan, China), ethanol (95%), and potassium bromide were purchased from Sinopharm Chemical Reagent Co., Ltd. (Shanghai, China). Linaoxin (Tonghua Boxiang Pharmaceutical Co., Ltd., Tonghua, China) [32], Levofloxacin (Jingxin Pharmaceutical Co., Ltd., Shaoxing, China) [33], and Lingzhi spore powder (namely *Ganoderma lucidum* spore powder, Senyu Pharmaceutical Co., Ltd., Osaka, Japan) and their active pharmaceutical ingredients (APIs) were purchased directly from pharmacies in Huainan, China.

### 3.2. Preparation of Pregelatinized Starch-Microcrystalline Cellulose Co-Processed Materials

A 1 kg mixture of corn starch and MCC at varying ratios was dispersed in 4.8 kg of purified water and stirred to homogeneity. The mixture was then processed twice using a colloid mill. Subsequently, 3.2 kg of anhydrous ethanol was added, and the mixture was placed in a glass reaction vessel. The reaction was conducted at 80 °C for 90 min with continuous stirring at 100 rpm. [11], followed by centrifugation at 1000 rpm to remove the liquid phase. The gelatinization of corn starch was confirmed through structural analysis using both scanning electron microscopy (SEM) and polarized light microscopy. Following gelatinization in water, corn starch formed a highly viscous gel. Utilizing the insolubility of pregelatinized starch in ethanol, the gelatinized gel was transformed into a suspension. Subsequently, facile solid-liquid separation was enabled via simple centrifugation. Promising feasibility and potential for industrial-scale production were demonstrated by this approach.

The resulting material was then dispersed in 75% (*v*/*v*) ethanol at room temperature with continuous stirring at 100 rpm for 30 min, followed by another centrifugation step at 1000 rpm to remove the liquid. The solid was then dried in a 105 °C forced-air drying oven until the loss on drying was between 3.0% and 8.0%, milled, and passed through an 80-mesh sieve to obtain the pregelatinized starch-microcrystalline cellulose co-processed material (PS-MCC), which was then stored for subsequent use. The ethanol residue in the coproduct prepared by this method was less than 0.5%. The prepared samples were designated as PS, PS-MCC-91, PS-MCC-82, PS-MCC-73, and PS-MCC-55, corresponding to corn starch and MCC mass ratios of 10:0, 9:1, 8:2, 7:3, and 5:5, respectively. All preparation parameters, including temperature, time, stirring speed, and centrifugation speed for liquid removal, were kept consistent except for the varying ratios.

### 3.3. Microscopic Morphology—Scanning Electron Microscopy (SEM)

The sample powder was evenly dispersed on an aluminum stub and sputtered onto a gold target using a vacuum coater at 1200 V and 20 mA for 105 s (approximately 10 nm thick). Observation was performed using an SU8100 electron microscope with the secondary electron upper probe (SE-U) selected, with an accelerating voltage of 5 kV–15 kV, a working distance of 6 mm, a sample stage tilt of 0°, and slow scan mode selected (scan speed 7, resolution 1024 × 884) [34].

### 3.4. Fourier Transform Infrared Spectroscopy (FTIR)

The sample dried at 105 °C for 2 h was uniformly mixed with potassium bromide at a mass ratio of 1:100. A small portion of the mixture was placed into a circular mold and compressed into a transparent pellet using a manual hydraulic press under 10 MPa pressure. The transparent pellet was then subjected to scanning in the beam path of an Antaris II Fourier transform infrared spectrometer, with the spectral wavelength range set at 500–4500 cm^−1^ [35].

### 3.5. X-Ray Diffraction (XRD)

The sample was mounted on an X-ray diffractometer and scanned over a 2θ range of 0–50° at a scanning rate of 2.5°/min. Cu Kα radiation (λ = 1.5406 Å) was used as the X-ray source, with the X-ray tube operating at 30 kV and 10 mA. The measurements were conducted under vacuum conditions ranging from 10^−6^ to 10^−7^ Torr [36].

### 3.6. Particle Size and Particle Size Distribution

The particle size of the samples was determined using dry dispersion. A 10 g sample was weighed and introduced into a Malvern laser particle size analyzer. The particle size distribution span (S) was calculated using Formula (1) according to the method in reference [37], and each sample was measured in triplicate.

(1) $$Span=\frac{\left(D_{90}-D_{10}\right)}{D_{50}}$$

Among them, D_90_ represents the particle size corresponding to when the cumulative particle size distribution percentage from small to large in the sample particle size results reaches 90%. D_10_ and D_50_ are calculated based on the same principle.

### 3.7. Powder Density and Carr’s Index

Approximately 30 g (M1) of the sample was weighed and transferred to a 100 mL graduated cylinder. The initial volume occupied by the powder was recorded as V1. The cylinder was then sealed with tape until the powder volume stopped changing, and the final volume was recorded as V2. The bulk density, tapped density, and Carr’s index were calculated using the following formulas [17].

(2) $$Bulk\;Density\;(g/{cm}^{3})=\frac{M_{1}}{V_{1}}$$

(3) $$Tapped\;Density\;(g/{cm}^{3})=\frac{M_{1}}{V_{2}}$$

(4) $$Carr\;Index\left(\%\right)=\frac{Tapped\;Density-Bulk\;Density}{Tapped\;Density}\times 100\%$$

### 3.8. Swelling Degree

A precisely weighed 6.0 g sample was uniformly distributed at the bottom of a 100 mL beaker. Subsequently, 50 mL of distilled water was added, and the mixture was stirred clockwise until a homogeneous transparent solution was obtained. The system was then maintained at ambient temperature (25 ± 1 °C) under quiescent conditions. Throughout the 120 min test period, the beaker was intermittently tapped to facilitate bubble removal from the sample matrix. The final volume was recorded at the designated time point, with the mean value of maximum and minimum height measurements being adopted in cases of irregular liquid surface formation [38].

### 3.9. Substances Soluble in Water

The method for determining the dissolved matter in water of PS in the 2020 edition of the Chinese Pharmacopoeia was used with slight modifications [39]; 2 g of sample (on a dry basis) was added to 50 mL of water, stirred for 10 min, and centrifuged (3000 rpm, 15 min); 25 mL of the supernatant was transferred to an evaporating dish, evaporated in a water bath, and dried at 120 °C for 4 h. The dissolved matter in water was calculated according to Formula (5).

(5) $$Water-soluble\;substances\left(\%\right)=\frac{\left(B-A\right)\times 10000}{25\times S\times W\times \left(100-C\right)}\times 100\%$$

where A represents the initial weight of the evaporating dish (g); B signifies the weight of the evaporating dish after drying (g); C denotes the loss on drying of the sample (%); S is the sample weight (g); and W is the mass ratio of corn starch in the combination of corn starch and MCC.

### 3.10. Determination of Tensile Strength

Tablets were prepared from each excipient sample using a multi-functional tablet press and stored in a desiccator for 24 h to allow for complete elastic recovery. Tablet hardness F (N), tablet diameter D (7 mm), and tablet thickness L (2.5 mm) were then measured, and tensile strength (TS) was calculated using Formula (6) [29]. Furthermore, the tableting performance of the co-processed material and its corresponding physical mixture in levofloxacin formulations (formulation details in Table 5) was investigated.

(6) $$TS\left(Mpa\right)=\frac{2F}{\pi DL}$$

### 3.11. Dilution Capacity Determination

Using the APIs of Linaoxin (a poorly compressible, lipid-soluble drug) and Lingzhi spore powder (a poorly compressible, highly oily botanical material) as model drugs, formulations were prepared by combining each drug with the co-processed excipient PS-MCC-73 at gradient drug loadings (30–50%, *w*/*w*). Magnesium stearate and other excipients were added, and the blends were uniformly mixed. Tablets were subsequently compressed (*n* = 3 batches; 100 tablets per batch). A minimum tablet hardness requirement of >25 N was set. The maximum drug loading that could be accommodated under this hardness requirement was defined as the dilution capacity of PS-MCC-73 for the respective model drug [40,41].

### 3.12. Biological Experiments

The *Escherichia coli* colony was added to LB medium and cultured in a shaking table for 3 h. Then, 100 mL of each medium was added with 0.1 g, 0.07 g, and 0.05 g of the PS-MCC-73 and their corresponding mixture, respectively. The medium was placed in a shaking table together with the control group and cultured overnight for 8 h. The absorbance of *E. coli* was detected at 600 nm using a spectrophotometer.

### 3.13. Statistical Analysis

All experiments were performed in triplicate, and the results were presented as mean ± standard error. Data were analyzed using SPSS 25.0 software with analysis of variance (ANOVA). Different lowercase letters (a–e) indicated significant differences. In addition, the two-sample *t*-test was implemented using R software 4.4.1 [42], and a *p*-value < 0.05 was considered statistically significant.

## Figures and Tables

**Table 1:** Particle size distribution of PS-MCC with different proportions of MCC.

Samples	D_10_ (µm)	D_50_ (µm)	D_90_ (µm)	Span
PS	30.32 ± 1.56 ^a^	113.90 ± 2.18 ^a^	196.77 ± 2.97 ^a^	1.46 ± 0.04 ^c^
PS-MCC-91	31.46 ± 1.56 ^a^	112.32 ± 2.03 ^a^	195.03 ± 3.04 ^a^	1.46 ± 0.03 ^c^
PS-MCC-82	30.74 ± 2.11 ^a^	109.33 ± 1.11 ^a^	193.30 ± 3.69 ^a^	1.49 ± 0.04 ^c^
PS-MCC-73	26.37 ± 0.78 ^b^	95.04 ± 1.37 ^b^	188.13 ± 3.94 ^b^	1.70 ± 0.02 ^b^
PS-MCC-55	18.02 ± 2.71 ^c^	65.49 ± 2.18 ^c^	168.23 ± 3.04 ^c^	2.30 ± 0.10 ^a^

Note: Data are expressed as mean ± SD, Different lowercase letters (a–c) indicate significant differences between different samples, *p* < 0.05.

**Table 2:** Powder properties of PS-MCC with different proportions of MCC.

Samples	Bulk Density (g/cm^3^)	Tapped Density (g/cm^3^)	Carr Index (%)
PS	0.6885 ± 0.016 ^a^	0.8771 ± 0.010 ^a^	22.25 ± 1.53% ^c^
PS-MCC-91	0.6602 ± 0.006 ^a^	0.8838 ± 0.004 ^a^	25.30 ± 0.44% ^b^
PS-MCC-82	0.6209 ± 0.012 ^b^	0.8498 ± 0.014 ^b^	26.94 ± 0.62% ^b^
PS-MCC-73	0.5683 ± 0.017 ^c^	0.7899 ± 0.006 ^c^	28.06 ± 0.66% ^b^
PS-MCC-55	0.4160 ± 0.008 ^d^	0.6061 ± 0.026 ^d^	31.30 ± 1.06% ^a^

Note: Data are expressed as mean ± SD, Different lowercase letters (a–d) indicate significant differences between different samples, *p* < 0.05.

**Table 3:** Swelling and water-soluble substances of PS-MCC with different proportions of MCC.

Sample	Water-Soluble Substances (%)	Swelling (mL)
PS	6.48 ± 0.23 ^a^	53.67 ± 0.17 ^a^
PS-MCC-91	5.63 ± 0.09 ^b^	52.17 ± 0.17 ^b^
PS-MCC-82	3.62 ± 0.10 ^c^	50.83 ± 0.44 ^b^
PS-MCC-73	3.12 ± 0.05 ^d^	47.50 ± 1.32 ^c^
PS-MCC-55	2.14 ± 0.04 ^e^	41.50 ± 0.50 ^d^

Note: Data are expressed as mean ± SD, Different lowercase letters (a–e) indicate significant differences between different samples, *p* < 0.05.

**Table 4:** Tableting effect of PS-MCC-73 in different model drugs.

Active Pharmaceutical Ingredients (API)	Drug Loading Capacity (%)	Tablet Hardness (N)	Formulations
Linaoxin	50	28.66 ± 0.56 ^b^	Linaoxin (40%, 45%, 50%), PC-MCC-73 (58%, 53%, 48%) Sodium Starch Glycolate (1%), Magnesium Stearate (1%)
	45	30.32 ± 0.41 ^b^	
	40	32.13 ± 0.18 ^a^	
Lingzhi spore powder	40	26.99 ± 0.31 ^b^	Lingzhi spore powder (30%, 35%, 40%), PC-MCC-73 (49%, 44%, 39%), Betacyclodextrin (15%), Povidone K30 (5%), Magnesium Stearate (1%)
	35	31.93 ± 0.46 ^a^	
	30	33.22 ± 0.69 ^a^	

Note: Data are expressed as mean ± SD, Different lowercase letters (a,b) indicate significant differences between different samples, *p* < 0.05.

**Table 5:** Comparison of the components of levofloxacin formulations in which PS and MCC were replaced with PS-MCC-73 in this study.

Prescription 1		Prescription 2	
Designation	Composition (%)	Designation	Composition (%)
Levofloxacin	70%	Levofloxacin	70%
Microcrystalline Cellulose SH-102 (MCC SH-102)	6.30%	pregelatinized starch microcrystalline cellulose co-processed material (PS-MCC-73)	21%
Pregelatinized starch SH-YJ-H (PS SH-YJ-H)	14.70%		
Crospovidone SH-SL	4%	Crospovidone SH-SL	4%
Hydroxypropylcellulose SH-L (HPC SH-L)	4%	HPC SH-L	4%
Sodium stearyl fumarate SH-AF (SSF SH-AF)	1%	SSF SH-AF	1%

## Data Availability

The original contributions presented in this study are included in the article/Supplementary Material. Further inquiries can be directed to the corresponding author.

## Supplementary Materials

The following supporting information can be downloaded at https://www.mdpi.com/article/10.3390/ph18091389/s1, Figure S1: Microscope image of the co-processed PS-MCC. The blue circle marks the polarization cross, while the yellow circle shows that the polarization cross disappears on some particles (×400).

## Abbreviations

APIs, active pharmaceutical ingredients; FTIR, Fourier Transform Infrared Spectroscopy; HPC, Hydroxypropylcellulose; MCC, microcrystalline cellulose; PS, pregelatinized starch; PS-MCC, pregelatinized starch and microcrystalline cellulose co-processed material; SEM, Scanning Electron Microscopy; SSF, Sodium stearyl fumarate; TS, tensile strength; XRD, X-ray Diffraction.
