# Supplementary material for: Study on the Physical Properties and Application of a Novel Pharmaceutical Excipient Made from Starch and Cellulose Co-Processing
Source: Pharmaceuticals (Basel). 2025 Sep 17;18(9):1389. doi: 10.3390/ph18091389 (PMC12472847; doi:10.3390/ph18091389)
Supplement: Supplementary file 1 [file pharmaceuticals-18-01389-s001.zip › pharmaceuticals-3734760-supplementary.pdf]

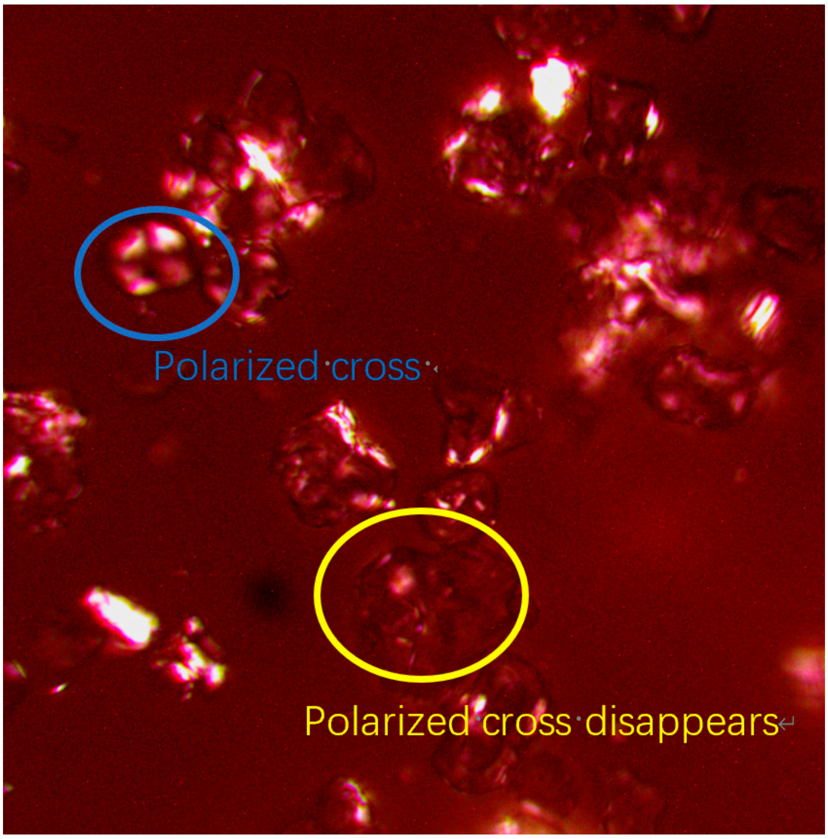

Figure S1 Microscope image of the co-processed PS-MCC. The blue circle marks the polarization cross, while the yellow circle shows that the polarization cross disappears on some particles (X400).
